# Patient and health system delays before registration among migrant patients with tuberculosis who were transferred out in China

**DOI:** 10.1186/s12913-018-3583-y

**Published:** 2018-10-19

**Authors:** Tao Li, Hui Zhang, Hemant Deepak Shewade, Kyaw Thu Soe, Lixia Wang, Xin Du

**Affiliations:** 1 National Center for Tuberculosis Control and Prevention, China CDC, No. 155 Changbai Road, Changping District, Beijing, 102206 China; 2 International Union Against Tuberculosis and Lung Disease (The Union), South-East Asian Office, Delhi, India; 3 Department of Medical Research (PyinOoLwin Branch), Ministry of Health and Sports, PyinOoLwin, Myanmar

**Keywords:** Patient delay, Health system delay, Diagnosis delay, Migrant, Transferred out, China, Operational research

## Abstract

**Background:**

Early diagnosis and treatment is vital for effective tuberculosis (TB) management especially among migrant populations who are a vulnerable group. We aimed to study factors associated with delay before registration at country level among registered migrant TB patients in China (2014–15) who were transferred out (during treatment) through web-based TB information management system (TBIMS).

**Methods:**

This was a cross sectional study involving review of TBIMS data. Delays (in days) were classified as follows: patient delay (from symptom onset to first doctor visit), health system delay (from first doctor visit to treatment initiation, divided into health system diagnosis and treatment delay before and after date of diagnosis respectively), diagnosis delay (from symptom onset to diagnosis) and total delay (from symptom onset to treatment initiation). Linear regression was used to build a predictive model (forward stepwise) for the socio-demographic, clinical and health system related factors associated with delay: one model for each type of delay. Delays were log transformed and included in the model.

**Results:**

The median (IQR) patient delay, health system delay and total delay was 16 (6, 34), two (0, 6) and 22 (11, 41) days respectively. Factors associated with long patient, diagnosis and total delay were: female gender, age ≥ 65 years, sputum smear positive pulmonary TB and registration at referral hospital. Treatment initiation delay was significantly higher among those registered in referral hospitals, unemployed and previously treated. Among migrant patients having permanent residence out of province, health system diagnosis delay was significantly higher while treatment initiation delay after diagnosis was significantly lower when compared to patients having permanent residence within the prefecture.

**Conclusion:**

Among migrant population with TB, patient delay contributed to the total delay. The factors identified including the need for improved coordination between referral hospitals and national programme have to be addressed if China has to end TB.

**Electronic supplementary material:**

The online version of this article (10.1186/s12913-018-3583-y) contains supplementary material, which is available to authorized users.

## Background

Globally, tuberculosis (TB) is the leading cause of death from infectious diseases. There were an estimated 10.4 million new TB patients in 2016, and 1.7 million died from the disease [1]. Early diagnosis and treatment is vital for effective TB management and is emphasized in World Health Organization (WHO) ‘The End TB strategy’ [2]. Delays in seeking diagnosis and treatment can result in severe clinical presentation at treatment initiation and unfavourable outcomes [3, 4].

China is listed as one of the 30 TB high burden countries, and has the third largest number of cases all over the world, with an estimated 895,000 (766000–1,030,000) incident cases in 2016 [1].^.^ Migrant TB populations are always considered as important vulnerable groups in TB control [5]. In China, migrant populations were mostly internal migrants than cross-border immigrants. Internal migrants (henceforth called as migrants) account for one-fifth of the whole population [6, 7]. In 2010, 29,924 new migrant patients with smear positive pulmonary TB (PTB) were registered, accounting for 7% of total new smear positive cases nationwide [8].

Globally, time delays and factors associated with delays among TB patients (among migrants or local residents) has been studied extensively [9–13]. Migrants have many specific characteristics such as instability, low income, poor living, and working condition which could obviously influence on their health seeking behavior and cause longer delay than general population [5, 14–18]. The inequality of social insurance coverage between local residents and migrant populations can further aggravate the delays [19–21].

Programmatically, two possible reasons for poor TB treatment outcomes among migrants are i) patient and health system delays before registration for treatment ii) high transfer outs during treatment which may contribute to treatment non evaluation. To address non-evaluation among transfer-outs, China has implemented web-based transfer-out using TB information management system (TBIMS) [22].

There have been many studies from China (among migrants and non-migrants) on delays in TB diagnosis and treatment [11, 12, 23–25]. Few focused on migrant population, none of them interpreted all stages of delays systematically and none were based on national level data. Furthermore, there was no study specifically addressing transferred-out migrant patients with TB. This subgroup of patient among migrant TB has higher risk of unfavourable outcomes. At the time of patient registration, the TBIMS systematically collects information on symptom onset and first doctor visit along with date of diagnosis and treatment initiation. It also collects information on permanent residence. This provides us an opportunity to study risk factors for delay at country level among migrant TB patients that were transferred out during treatment in China. The findings on extent of treatment non evaluation among transferred out migrant TB patients and its risk factors will be published elsewhere.

## Methods

### Study design

This is a cross sectional study involving review of TBIMS data.

### Setting

#### General setting

China is the world’s most populous country with a population of over 1.4 billion [1]. The sub-national administrative division consists of province or regions (*n* = 34), prefectures (*n* = 333) and counties (> 3000).

The National center for tuberculosis control and prevention (NCTB) belongs to Chinese center for disease control and prevention and is in charge of National TB programme. TB management units are established at provincial, prefecture and county levels (basic management units (BMU) at county level), including independent TB dispensaries, TB control divisions within local centers for disease control and prevention and health facilities designated by health authorities. TB diagnostic facilities are centralized at the county level and rarely also available at township level (below county). At BMUs, diagnosed patients are registered and initiated on directly observed therapy-short course (DOTS). They are assisted by township clinics and village health workers. Anti-tuberculosis drugs and needful examinations are provided without charge. Similar to a BMU, some regional referral hospitals also take patient management responsibility and register patients. Unlike in BMUs, the drugs and examinations are usually not free in referral hospitals. The payment could be reimbursed by social insurances but migrant population often have many barriers to get reimbursed [26].

#### China web-based TBIMS

**Fig. 1:**
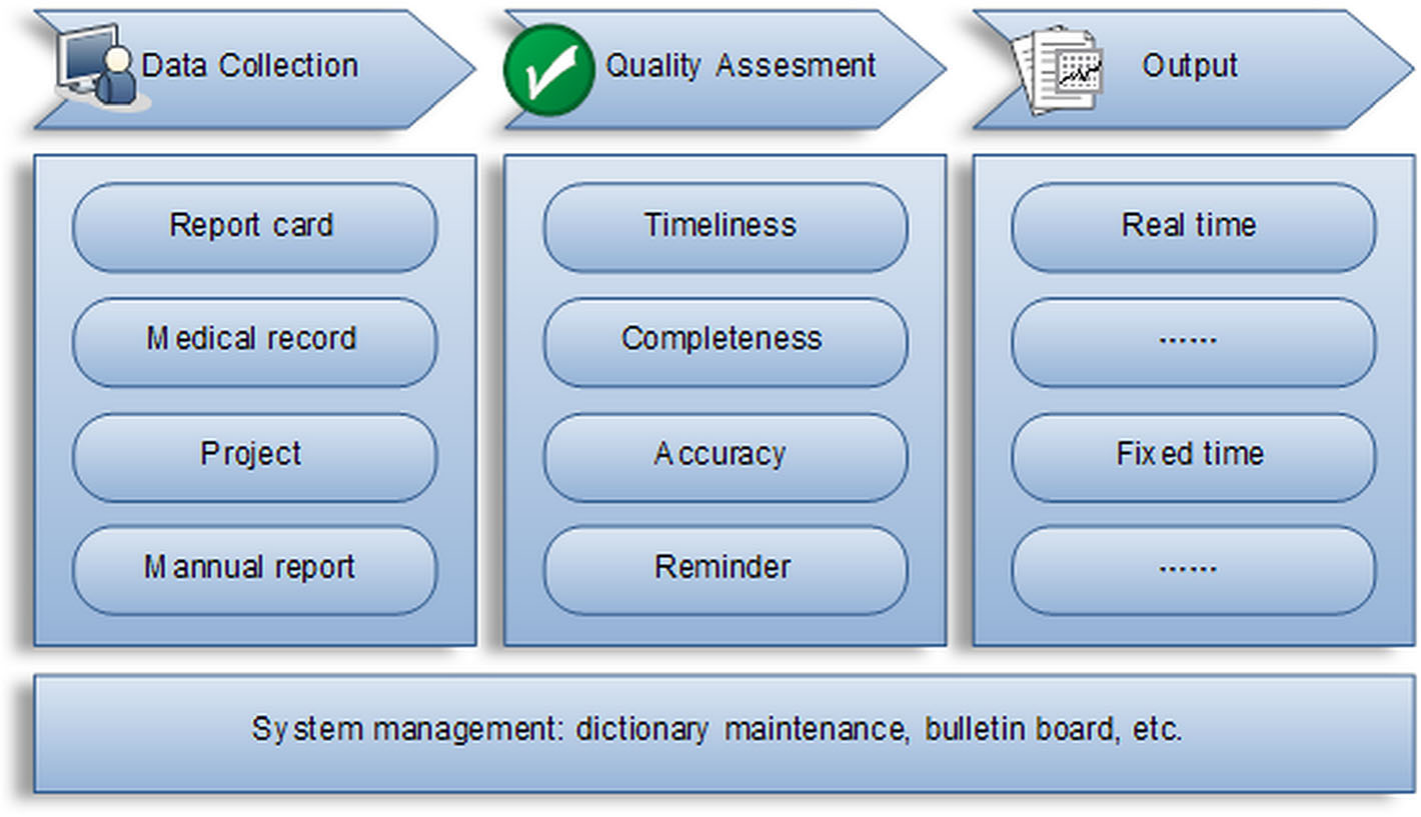
Diagram depicting functions of electronic TB information management system (TBIMS), China

Patients are registered for treatment in the web-based TBIMS. In January 2005, the Ministry of Health of China launched the first version of TBIMS. The second version of TBIMS was launched in April 2009. The transfer-in/out module was added in this update. The function of TBIMS is divided into 4 groups: data collection, quality assessment, and output and system management [27] (Fig. 1).

TBIMS routinely collects individual patients’ information as well as national TB programme (NTP) activities. Patients’ standard medical records are filled by doctors and entered into TBIMS by NTP health care workers, including all diagnosis, treatment, follow-up examinations, outcome and DOTS management information. NTP activities that are updated in TBIMS include training, supervision, meeting and health education. All data are restored in a centralized national data center run by China center for disease control. Individual cases and statistical reports can be generated, viewed, edited and extracted for patients care and programme management.

The transfer of patients with TB is implemented by BMU through TBIMS and the transfer-in BMU will be noticed in the system to trace the patient. If the patients were traced successfully, the transfer-in BMU will take over patients’ management, and update the outcome in TBIMS when patients finished their treatment.

### Study population and period

All migrant patients with TB registered in China during 2014–15 and transferred-out using web-based TBIMS anytime during their treatment were the study population.

**Migrant patient with TB** refers to those who were diagnosed as TB and registered at county BMU/referral hospital, were from another BMU, and stayed for less than 6 months within that county at the time of registration. Migrant status at registration is routinely captured in the TBIMS.

### Data collection

Secondary data was extracted from electronic TBIMS in Microsoft Excel (Microsoft, Redmond, WA, USA). Socio-demographic (age, gender, occupation and permanent residence of patient), clinical (TB classification, treatment category and HIV status) variables and BMU name were collected at the time of registration. In addition the following dates were also collected: symptom onset, first doctor visit, diagnosis, registration, treatment initiation.

### Data management and analysis

Database was constructed, cleaned and analyzed with Microsoft Excel (Microsoft, Redmond, WA, USA). Adjusted analysis was done using STATA (version 12.1, copyright 1985–2011 Stata Corp LP USA).

#### Derived variables

**Fig. 2:**
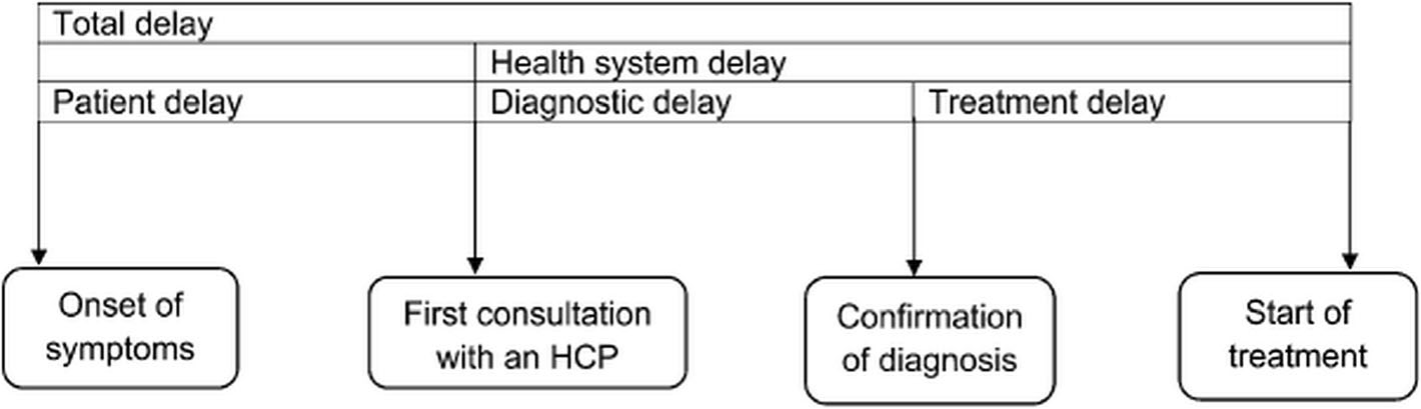
Conceptual framework on definitions of delay before treatment initiation among migrant TB patients that were transfer out using web-based TBIMS in China (2014–15)* [29]. *Reprinted with permission of the International Union Against Tuberculosis and Lung Disease. Copyright © The Union [29]. HCP – Health Care Providers

‘Whether registered at referral hospital’ was derived based on BMU name. Delays were calculated from different dates: patient delay (from symptom onset to first doctor visit), health system delay (from first doctor visit to treatment initiation, divided into health system diagnosis delay and treatment delay before and after date of diagnosis respectively), diagnosis delay (from symptom onset to diagnosis) and total delay (from symptom onset to treatment initiation) (Fig. 2).

#### Data analysis

Median and interquartile range (IQR) were used to summarize delays. Linear regression was used to build a predictive model (forward stepwise) for factors associated with delay: one model for each type of delay. Log transformation of delay variable (as it was not normally distributed) was done and included as outcome variable in each model. Age, gender and variables with unadjusted *p* value < 0.20 were considered in the models. First, the variable with lowest unadjusted p value was added followed by the variable with the next lowest unadjusted p value. At each step, the variable was retained if the likelihood ratio test was significant (*p* < 0.05). Beta coefficients (0.95 CI) were used to summarize (infer) the association of the variables included in the final model with delay.

## Results

There were 7284 patients. Various delays stratified by socio-demographic, clinical and health system level characteristics have been summarized in Tables 1 and 2. Because of missing dates, a total of 22 patient records were excluded and 7262 records were including in building the models.

**Table 1:** Patient and health system delay before registration among transferred out migrant patients with TB^a^, stratified by their socio-demographic characteristics, China, 2014–2015

Characteristics	Total patients (*n* = 7284)	Patient delay (*n* = 7275)	Health system diagnosis delay (*n* = 7283)	Health system treatment delay (*n* = 7270)	Diagnosis delay (*n* = 7269)	Health system delay (*n* = 7275)	Total delay (*n* = 7262)
	N (%)	Median (IQR)	Median (IQR)	Median (IQR)	Median (IQR)	Median (IQR)	Median (IQR)
Total	7284 (100)	16(6,34)	1 (0, 5)	0(0,0)	21(10,40)	2(0,6)	22(11,41)
Age group (in years)							
< 15	33 (0.5)	14(5,30)	2(0,3)	0(0,1)	19(10,32)	2(1,3)	19(10,32)
15–44	4261 (58.5)	15(4,33)	1(0,6)	0(0,0)	20(9,40)	2(0,6)	21(10,41)
45–64	2126 (29.2)	19(7,35)	2(0,5)	0(0,0)	23(11,42)	2(0,5)	23(12,43)
> =65	864 (11.8)	18(8,34)	2(0,5)	0(0,0)	23(12,39)	2(0,5)	23(12,40)
Gender							
Male	5107 (70.1)	15(5,33)	1(0,5)	0(0,0)	21(10,39)	2(0,5)	21(10,40)
Female	2177 (29.9)	17(6,37)	2(0,5)	0(0,0)	23(11,44)	2(0,6)	23(12,44)
Occupation							
Studying	492 (6.8)	9(2,26)	2(0,8)	0(0,1)	17(8,31)	3(1,9)	18(8,32)
Farmers and herdsmen	2321 (31.8)	22(7,37)	2(0,4)	0(0,0)	27(12,42)	2(0,5)	28(12,42)
Semi-skilled employee	107 (1.5)	10(1,31)	3(0,11)	0(0,0)	21(7,42)	3(0,11)	21(7,42)
Salaried employee	1294 (17.7)	14(5,32)	1(0,5)	0(0,0)	20(9,39)	1(0,5)	20(9,39)
Non-salaried employee	290 (4.0)	14(4,37)	1(0,4)	0(0,0)	20(10,42)	2(0,6)	20(10,43)
Unemployed	2328 (32.0)	14(5,33)	1(0,6)	0(0,1)	19(10,40)	2(0,6)	20(11,40)
Others	452 (6.2)	20(8,42)	1(0,5)	0(0,0)	27(13,55)	2(0,6)	28(14,59)
Permanent residence							
Within prefecture	4871 (66.9)	16(7,32)	2(0,5)	0(0,0)	21(11,38)	2(0,5)	21(11,38)
Within province	557 (7.6)	19(5,53)	1(0,4)	0(0,0)	26(10,60)	1(0,5)	26(10,62)
Out of province	1856 (25.5)	15(3,37)	1(0,6)	0(0,0)	22(9,48)	1(0,7)	22(9,49)

Few dates missing for some patients, hence total assessed for each type of delay may not be 7284

*TB* tuberculosis*, PTB* pulmonary tuberculosis*, IQR* interquartile range

^a^registered in web-based TB information management system

**Table 2:** Patient and health system delay before registration among transferred out migrant patients with TB^a^, stratified by their clinical and programmatic characteristics, China, 2014–2015

Characteristics	Total patients (*n* = 7284)	Patient delay (*n* = 7275)	Health system diagnosis delay (*n* = 7283)	Health system treatment delay (*n* = 7270)	Diagnosis delay (*n* = 7269)	Health system delay (*n* = 7275)	Total delay (*n* = 7262)
	N (%)	Median (IQR)	Median (IQR)	Median (IQR)	Median (IQR)	Median (IQR)	Median (IQR)
Total	7284 (100)	16(6,34)	1 (0, 5)	0(0,0)	21(10,40)	2(0,6)	22(11,41)
Classification							
PTB smear positive	2440 (33.5)	20(7,49)	1(0,4)	0(0,0)	24(11,57)	2(0,5)	25(11,59)
PTB smear negative	4324 (59.4)	15(4,32)	2(0,6)	0(0,0)	20(10,37)	2(0,6)	21(10,38)
PTB smear status unknown	34 (0.5)	22(7,47)	3(1,6)	0(0,0)	32(14,73)	4(1,8)	32(22,73)
Pleurisy	483 (6.6)	15(7,30)	2(0,6)	0(0,1)	20(12,32)	2(1,6)	20(13,32)
EPTB	3 (< 0.1)	25(17,74)	0(0,0)	0(0,1)	25(17,74)	0(0,1)	25(18,74)
Category							
New	6915 (94.9)	16(5,34)	1(0,5)	0(0,0)	21(10,40)	2(0,6)	22(11,41)
Retreated	369 (5.1)	19(8,41)	2(0,4)	0(0,1)	23(12,46)	2(0,5)	23(13,48)
HIV							
Positive	9 (0.1)	31(20,57)	2(0,10)	0(0,0)	44(29,59)	2(0,11)	44(29,59)
Negative	2864 (39.3)	14(6,33)	2(1,7)	0(0,1)	20(11,40)	3(1,8)	21(12,41)
Unknown^b^	4411 (60.6)	18(6,35)	1(0,4)	0(0,0)	22(10,40)	1(0,4)	23(10,41)
Registered in referral hospital							
Yes	4153 (57.0)	17(7,33)	2(1,5)	0(0,0)	22(11,38)	2(1,6)	23(11,39)
No	3131 (43.0)	15(4,36)	1(0,5)	0(0,0)	21(9,44)	1(0,6)	21(10,46)

Few dates missing for some patients, hence total assessed for each type of delay may not be 7284

*TB* tuberculosis*, PTB* pulmonary tuberculosis*, EPTB* extrapulmonary tuberculosis*, HIV* human immunodeficiency virus*, IQR* interquartile range

^a^registered in web-based TB information management system

^b^TB examinations were routinely carried out in all new or follow up HIV/AIDS patients nationwide while TB patients were screened with HIV tests only in selected high HIV epidemic counties

### Patient delay

The median (IQR) patient delay was 16 (6, 34) days. Final model for independent predictors for patient delay is shown in Table 3. Female gender (β = 0.11, *p* = 0.003), and patients with sputum smear positive pulmonary TB (β = 0.33, *p* < 0.001) were independent predictors for longer delay. Age between 15 and 44 years (β = − 0.23, *p* < 0.001) and patients registered in programme BMU (β = − 0.20, *p* < 0.001) were independent predictors for shorter delay. Though included in the final model, migrant patient permanent residence was not an independent predictor. Occupation, treatment category and HIV status were excluded by the model.

**Table 3:** Linear regression for independent predictors of patient delay before registration among transferred out migrant patients with TB^a^, China, 2014–15^b^ (*N* = 7262)^c^

Variable	Β coefficient	95% CI	*P* value
Age group (in years)			
< 15	−0.30	− 0.82, 0.21	0.250
15–44	− 0.23	− 0.34, − 0.12	< 0.001^^^
45–64	0.03	− 0.09, 0.14	0.672
> =65	Ref	Ref	Ref
Gender			
Male	Ref	Ref	Ref
Female	0.11	0.04, 0.19	0.003^^^
Classification			
PTB smear positive	0.33	0.19, 0.48	< 0.001^^^
PTB smear negative	−0.04	−0.18, 0.10	0.572
PTB smear status unknown	0.37	−0.14, 0.89	0.157
Pleurisy	Ref	Ref	Ref
EPTB	1.02	−0.66, 2.70	0.235
Registered at referral hospital			
Yes	Ref	Ref	Ref
No	−0.20	−0.27, − 0.13	< 0.001^^^

*TB* tuberculosis*, PTB* pulmonary tuberculosis*, EPTB* extrapulmonary tuberculosis

^a^registered in web-based TB information management system

^b^logarithmic transformation of delay variable done as it was not normally distributed, model building was done by stepwise (forward method) method and the final model has been presented; occupation, permanent residence, treatment category and HIV status, though considered, were excluded by the model (likelihood ratio test)

*F* stat = 20.8; Probability > *F* = < 0.001

^c^of 7284, twenty two patient records were excluded because of missing information on dates to calculate delay

^^^significant *p*-value < 0.05

### Health system delay

The median (IQR) health system delay was two (0, 6) days. Final model for independent predictors for health system delay is shown in Table 4. Patients with unknown sputum smear status (β = 0.57, *p* = 0.003) was independent predictor for longer delay, while unknown HIV status (β = − 0.40, *p* < 0.001) was independent predictor for shorter delay. Age group, gender, occupation, permanent residence, treatment category and registration at referral hospital were excluded by model.

**Table 4:** Linear regression for independent predictors of health system delay before registration among transferred out migrant patients with TB^a^, China, 2014–15^b^ (*N* = 7262)^c^

Variable	Β coefficient	95% CI	*P* value
Classification			
PTB smear positive	−0.10	−0.21, 0.002	0.056
PTB smear negative	0.02	−0.08, 0.13	0.631
PTB smear status unknown	0.57	0.20, 0.95	0.003^^^
Pleurisy	Ref	Ref	Ref
EPTB	−0.82	−2.04,0.41	0.193
HIV			
Positive	−0.04	−0.75, 0.67	0.911
Negative	Ref	Ref	Ref
Unknown	−0.40	−0.45, − 0.35	< 0.001^^^

*TB* tuberculosis*, PTB* pulmonary tuberculosis*, EPTB* extrapulmonary tuberculosis*, HIV* human immunodeficiency virus

^a^registered in web-based TB information management system

^b^logarithmic transformation of delay variable done as it was not normally distributed, model building was done by stepwise (forward method) method and the final model has been presented; age group, gender, occupation, permanent residence, treatment category and registration at referral hospital, though considered, were excluded by the model (likelihood ratio test)

*F* stat for model = 43.6; Probability > *F* = < 0.001

^c^of 7284, twenty two patient records were excluded because of missing information on dates to calculate delay

^*^*^significant *p*-value < 0.05

### Health system diagnosis delay

The median (IQR) health system diagnosis delay was one (0, 5) day. Final model for independent predictors for health system diagnosis delay is shown in Table 5. Independent predictors of health system diagnosis delay were similar to predictors of overall health system delay, but in addition sputum smear positive TB (β = − 0.16, *p* < 0.003) was also independent predictor for shorter delay and permanent residence being out of province (β = 0.13, *p* < 0.001) was independent predictor for longer delay. Age group, gender, occupation, treatment category and registration at referral hospital were excluded by the model.

**Table 5:** Linear regression for independent predictors of health system diagnosis delay before registration among transferred out migrant patients with TB^a^, China, 2014–15^b^ (*N* = 7262)^c^

Variable	Β coefficient	95% CI	*P* value
Permanent residence			
Within prefecture	Ref	Ref	Ref
Within province	−0.004	− 0.10,0.09	0.932
Out of province	0.13	0.07,0.19	< 0.001^^^
Classification			
PTB smear positive	−0.16	−0.27-0.06	0.003^^^
PTB smear negative	0.01	−0.09,0.12	0.784
PTB smear status unknown	0.40	0.02,0.77	0.037^^^
Pleurisy	Ref	Ref	Ref
EPTB	−0.77	−1.99,0.45	0.218
HIV			
Positive	0.06	−0.64,0.77	0.864
Negative	Ref	Ref	Ref
Unknown	−0.33	−0.38,-0.28	< 0.001^^^

*TB* tuberculosis*, PTB* pulmonary tuberculosis*, EPTB* extrapulmonary tuberculosis*, HIV* human immunodeficiency virus

^a^registered in web-based TB information management system

^b^logarithmic transformation of delay variable done as it was not normally distributed, model building was done by stepwise (forward method) method and the final model has been presented; age group, gender, occupation, treatment category and registration at referral hospital, though considered, were excluded by the model (likelihood ratio test)

*F* stat for model = 26.8; Probability > *F* = < 0.001

^c^of 7284, twenty two patient records were excluded because of missing information on dates to calculate delay

^^^significant *p*-value < 0.05

### Health system treatment initiation delay

The median (IQR) health system treatment initiation delay was zero (0, 0) day. Final model for independent predictors for health system treatment initiation delay is shown in Table 6. Independent predictors of health system treatment initiation delay were similar to predictors of overall health system delay, but in addition permanent residence being out of province (β = − 0.03, *p* < 0.001), occupation being farmers/herdsmen (β = − 0.02, *p* = 0.013) and salaried employee (β = − 0.03, *p* = 0.002) and new TB patients (β = − 0.02, *p* = 0.014) were independent predictors for shorter delay. Age group, gender and registration at referral hospital were excluded by the model.

**Table 6:** Linear regression for independent predictors of health system treatment initiation delay before registration among transferred out migrant patients with TB^a^, China, 2014–15^b^ (*N* = 7262)^c^

Variable	Β coefficient	95% CI	*P* value
Occupation			
Studying	0.01	−0.01,0.04	0.370
Farmers and herdsmen	−0.02	−0.04,-0.004	0.013^^^
Semi-skilled employee	−0.003	−0.05,0.05	0.899
Salaried employee	−0.03	−0.05,-0.01	0.002^^^
Non-salaried employee	0.001	−0.03,0.03	0.970
Unemployed	Ref	Ref	Ref
Others	−0.01	−0.04,0.02	0.523
Permanent residence			
Within prefecture	Ref	Ref	Ref
Within province	−0.02	−0.04,0.01	0.134
Out of province	−0.03	−.0.05,-0.02	< 0.001^^^
Classification			
PTB smear positive	0.02	−0.002,0.05	0.071^^^
PTB smear negative	−0.01	−0.04,0.01	0.391
PTB smear status unknown	0.25	0.16,0.35	< 0.001^^^
Pleurisy	Ref	Ref	Ref
EPTB	−0.03	−0.33,0.27	0.830
Category			
New	−0.04	−0.07,-0.01	0.014^^^
Retreated	Ref	Ref	Ref
HIV			
Positive	−0.07	−0.24,0.10	0.425
Negative	Ref	Ref	Ref
Unknown	−0.34	−0.05,-0.02	< 0.001^^^

*TB* tuberculosis*, PTB* pulmonary tuberculosis*, EPTB* extrapulmonary tuberculosis*, HIV* human immunodeficiency virus

^a^registered in web-based TB information management system

^b^logarithmic transformation of delay variable done as it was not normally distributed, model building was done by stepwise (forward method) method and the final model has been presented; age group, gender and registration at referral hospital, though considered, were excluded by the model (likelihood ratio test)

*F* stat for model = 10.6; Probability > *F* = < 0.001

^c^of 7284, twenty two patient records were excluded because of missing information on dates to calculate delay

^*^*^significant *p*-value < 0.05

### Diagnosis delay

The median (IQR) diagnosis delay was 21 (10, 40) days. Final model for independent predictors for diagnosis delay is shown in Table 7. Independent predictors for diagnosis delay were similar to predictors of patient delay.

**Table 7:** Linear regression for independent predictors of diagnosis delay before registration among transferred out migrant patients with TB^a^, China, 2014–15^b^ (*N* = 7262)^c^

Variable	Β coefficient	95% CI	*P* value
Age group (in years)			
< 15	−0.26	−0.69,0.17	0.240
15–44	−0.10	−0.19,-0.01	0.037^^^
45–64	0.03	−0.07,0.13	0.560
> =65	Ref	Ref	Ref
Gender			
Male	Ref	Ref	Ref
Female	0.11	0.05,0.18	< 0.001^^^
Classification			
PTB smear positive	0.21	0.08,0.33	0.001^^^
PTB smear negative	−0.01	−0.13,0.10	0.814
PTB smear status unknown	0.44	0.01,0.87	0.047^^^
Pleurisy	Ref	Ref	Ref
EPTB	0.53	−0.88,1.95	0.462
Registered at referral hospital			
Yes	Ref	Ref	Ref
No	−0.06	−0.13,-0.003	0.037^^^

*TB* tuberculosis*, PTB* pulmonary tuberculosis*, EPTB* extrapulmonary tuberculosis

^a^registered in web-based TB information management system

^b^logarithmic transformation of delay variable done as it was not normally distributed, model building was done by stepwise (forward method) method and the final model has been presented; occupation, permanent residence, treatment category and HIV status, though considered, were excluded by the model (likelihood ratio test)

*F* stat for model = 9.28; Probability > *F* = < 0.001

^c^of 7284, twenty two patient records were excluded because of missing information on dates to calculate delay

^*^*^significant *p*-value < 0.05

### Total delay

The median (IQR) total delay was 22 (11, 41) days. Final model for independent predictors for total delay is shown in Table 8. Independent predictors for total delay were similar to predictors of patient delay.

**Table 8:** Linear regression for independent predictors of total delay before registration among transferred out migrant patients with TB^a^, China, 2014–15^b^ (*N* = 7262)^c^

Variable	Β coefficient	95% CI	*P* value
Age group (in years)			
< 15	−0.23	−0.65,0.20	0.300
15–44	−0.11	−0.20,-0.01	0.024^^^
45–64	0.03	−0.07,0.12	0.605
> =65	Ref	Ref	Ref
Gender			
Male	Ref	Ref	Ref
Female	0.11	0.05,0.18	< 0.001^^^
Classification			
PTB smear positive	0.21	0.09,0.33	0.001^^^
PTB smear negative	−0.02	−0.14,0.10	0.740
PTB smear status unknown	0.53	0.10,0.96	0.015^^^
Pleurisy	ref		
EPTB	0.54	0.85,1.93	0.445
HIV			
Positive	0.61	−0.19,1.41	0.134
Negative	Ref	Ref	Ref
Unknown	−0.04	−0.10,0.02	0.166
Registered at referral hospital			
Yes	Ref	Ref	Ref
No	−0.07	−0.13,-0.003	0.038^^^

*TB* tuberculosis*, PTB* pulmonary tuberculosis*, EPTB* extrapulmonary tuberculosis*, HIV* human immunodeficiency virus

^a^registered in web-based TB information management system

^b^logarithmic transformation of delay variable done as it was not normally distributed, model building was done by stepwise (forward method) method and the final model has been presented; occupation, permanent residence and treatment category, though considered, were excluded by the model (likelihood ratio test)

*F* stat for model = 9.1; Probability > *F* = < 0.001

^c^of 7284, twenty two patient records were excluded because of missing information on dates to calculate delay

^^^significant *p*-value < 0.05

### Summary of independent predictors

The independent predictors of each type of delay have been summarized in Additional file 1: Table S1. The positive or negative sign in brackets indicates the direction of association: positive sign means the factor is an independent predictor for longer delay and negative sign means the factor is an independent predictor for shorter delay when compared to reference.

## Discussion

This was the first country wide study from China looking at delay before registration among migrant TB patients. There were some key findings from this study. The median total delay was 22 days which was significantly contributed by median 16 days of patient delay, while health system delay was much shorter. Elderly age group, female gender, patients registered in referral hospitals and patients with pulmonary sputum smear positive TB were factors associated with longer patient delay, diagnosis delay and total delay. Patients registered in referral hospital or with previous treatment history had significant higher treatment initiation delay.

### Short patient and health system delays

Patient delay significantly contributed to total delay: this was similar to other studies in India, China and Asia [28–30]. The median total delay (22 days) and patient delay (16 days) were quite shorter compared to studies in other high TB burden countries [28, 30]. Migrant TB patients received proper diagnosis and management within very short duration of having any symptom of TB and almost immediately after visiting a health facility in China [31] as other general populations [32].

We speculate that this was due to four important reasons. Firstly, most destinations of migration were urban areas and had obviously better universal health coverage [6, 7]. We did not adjust for this factor (urban/rural) in our analysis. Secondly, it might be related to the annual health examination (including TB) especially among people working at industries or companies where people migrated and worked, having high chance of early diagnosis and treatment [33, 34]. Thirdly, patients probably benefitted from the public-public mix collaboration among different public health providers and TB designated facilities which was implemented after severe acute respiratory syndrome epidemic [35]. Fourth, there were very few private practitioners in China, most health providers were public hospitals. According to national law and regulation of infectious diseases control, all health facilities must report TB cases within 24 h after diagnosis and refer them to programme BMUs since 2004 [36, 37]. BMUs must trace these referred patients with TB and get them involved in programme management [22]. These mandatory requirements possibly shortened the health system diagnosis delay compared to historical studies before in China [38, 39]. On the other hand, providing free anti-tuberculosis drugs by programme BMUs reduced the health system treatment delay [22], which was also relatively shorter than most other low-middle income countries [4, 13, 40], while similar as in South Africa [41].

### Predictors of patient delay, diagnosis and total delay

Independent predictors for long patient delay, diagnosis delay and total delay were the same (elderly age group, female gender, patients registered in referral hospitals and patients with pulmonary sputum smear positive TB). It may be because patient delay contributed to most of the diagnosis and total delay.

Female migrant TB patients being associated with delays has also been reported elsewhere/before in China, India, and Asia [11, 12, 29]. Females might prefer self-treatment using home remedies to treat their TB like symptoms at home which might lead to delay in seeking care. Compared with pleurisy, patients with sputum smear positive were associated with delay. It might be that TB symptoms were relatively more severe among pleurisy patients [42]. This study also found that patients with 15–44 years of age were less likely to have delays than older age group. This finding contradicts with the finding from India where patients < 45 years of age were at higher risk for delay [43]. Patients who were registered by referral hospitals had longer delay when compared to programme BMUs. This could be explained by the fact that patients visited referral hospital after visiting other public facilities or after becoming sick.

### Predictors of health system delay

Among migrant patients having permanent residence out of province, health system diagnosis delay was significantly higher while treatment initiation delay was significantly lower when compared to migrant patients having permanent residence within the prefecture. This was intriguing. The former needs to be addressed by the programme.

Contrarily, sputum smear positive PTB in the model of patient delay was an independent predictor for longer delay but in the model of health system diagnosis delay was an independent predictor for shorter delay. It may that smear positive PTB patients were easier to be diagnosed for bacteriological evidence, more likely to be infectious and NTP paid more attention to them than TB pleurisy patients.

Previously treated patients had significantly higher treatment initiation delay which could be because of their previous bad experience of TB treatment, similar as reported elsewhere/before in India and China [13, 32]. Patient registered in referral hospital had significant higher treatment initiation delay after diagnosis due to procedural and interest reasons [44].

### Policy implications

Though programme is doing well in shortening patient delays when compared to other countries, however, these can be further reduced if we want to move towards ending TB [45]. Migrant populations were less likely to be covered by social insurance, which not only caused delays due to their health seeking behaviors, but also led to high risk of transfer-out during the treatment (programme records). In order to change this situation, firstly migrant population needs to be treated equally under universal health coverage. Providing subsidies to migrant patients with TB could effectively encourage them visit doctors and reduce the patient delay, and ensure them stay in the diagnosed place to complete treatment [46, 47].

Lack of knowledge of TB symptoms, poor policy awareness and stigma were the most common risk factors identified previously with patient delay which could be even more among elderly and women migrants [12, 28–30]. The programme should focus on health promotion activities among elderly and women migrants who are probably family numbers of migrant workers and have less access to work place interventions. In addition, active case finding in migrant population could also reduce the patient delay [9].

Around two-thirds of patients in our study were registered in referral hospitals and had delayed diagnosis and treatment initiation. China must consider ameliorating the implementation of NTP policies in referral hospitals. Referral hospitals have less motivation to involve patients in programme and complete the full course treatment because of free drugs and standardized follow-up examinations [44]. It is imperative to build efficient coordination mechanisms between referral hospitals and national programme. More studies need to be implemented to explore the mechanisms and activities which could eventually help reducing patient level, diagnosis and treatment initiation delay.

### Strengths and limitations

Few countries collect sufficiently disaggregated data on the health of migrants [5, 48] and this is a major strength of the study. Sustainable development goal number 17 includes two targets and associated indicators under the subheading of ‘data, monitoring and accountability’ which include mechanism to generate disaggregate data for specific subpopulations. Migrants are one such subpopulation. [1, 49] Second, the findings are representative of the situation in China as the study involved a national cohort of patients over 2 years without sampling. Third, there were minimal missing values, despite being a record review study.

This study could not include some important factors which influence delay, such as knowledge and attitude, type of health care provider visited, information on self-medications, smoking and alcohol use, family income, urban/rural residence and nutritional status. This information is not routinely collected by the TBIMS. The cross-sectional nature of data limited causal inference. Some speculations were discussed based on our experience of working within programme; future qualitative research is needed to further clarify these speculations. Finally, there are concerns regarding applying and interpreting the results of hypothesis testing in a log-transformed data on non-log-transformed data [50].

## Conclusion

Limitations notwithstanding, our findings indicate that delay before registration among migrant TB patients was lower than general population in China and globally. Patient delay contributed to the total delay. Elderly patients, females, those with sputum smear positive pulmonary TB and those registered in referral hospital had higher patient, diagnosis and total delay. Patients with permanent residence out of province had significantly higher delay in diagnosis after visiting a health facility. Patients registered in referral hospital also had significantly higher treatment initiation delay after diagnosis. If China has to attain the targets of ending TB by 2035 [45], these factors including improved coordination between referral hospitals and programme have to be addressed urgently.

## Additional file

Additional file 1: **Table S1.** Summary of independent predictors associated with different types of delays before registration among transferred out migrant patients with TB, China, 2014–15**.** All 5 types of delay (including Patient delay, Health system diagnosis delay, Health system treatment delay, Diagnosis delay and Health system delay) and predictors are listed together to show their association. (DOCX 21 kb)

## Abbreviations

BMU: Basic management unit
IQR: Interquartile range
NCTB: National Center for Tuberculosis Control and Prevention
PTB: Pulmonary TB
TB: Tuberculosis
TBIMS: TB information management system
WHO: World Health Organization
