# Supplementary material for: Patient and health system delays before registration among migrant patients with tuberculosis who were transferred out in China
Source: BMC Health Serv Res. 2018 Oct 19;18:786. doi: 10.1186/s12913-018-3583-y (PMC6194607; doi:10.1186/s12913-018-3583-y)
Supplement: Supplementary file 1 — Table S1. Summary of independent predictors associated with different types of delays before registration among transferred out migrant patients with TB, China, 2014–15. All 5 types of delay (including Patient delay, Health system diagnosis delay, Health system treatment delay, Diagnosis delay and Health system delay) and predictors are listed together to show their association. (DOCX 21 kb) [file 12913_2018_3583_MOESM1_ESM.docx]

**S1 Table.** Summary of independent predictors associated with different types of delays before registration among transferred out migrant patients with TB*, China, 2014-15

| **Variable** | | **Patient delay** | **Health system diagnosis delay** | **Health system treatment delay** | **Diagnosis delay** | **Health system delay** | **Total delay** |
| --- | --- | --- | --- | --- | --- | --- | --- |
| Age group (in years) | |  |  |  |  |  |  |
|  | <15 |  |  |  |  |  |  |
|  | 15-44 | Y(-) |  |  | Y(-) |  | Y(-) |
|  | 45-64 |  |  |  |  |  |  |
|  | >=65 | Ref |  |  | Ref |  | Ref |
| Gender | |  |  |  |  |  |  |
|  | Male | Ref |  |  | Ref |  | Ref |
|  | Female | Y(+) |  |  | Y(+) |  | Y(+) |
| Occupation | |  |  |  |  |  |  |
|  | Studying |  |  |  |  |  |  |
|  | Farmers and herdsmen |  |  | Y(-) |  |  |  |
|  | Semi-skilled employee |  |  |  |  |  |  |
|  | Salaried employee |  |  | Y(-) |  |  |  |
|  | Non-salaried employee |  |  |  |  |  |  |
|  | Unemployed |  |  | Ref |  |  |  |
|  | Others |  |  |  |  |  |  |
| Residency*** | |  |  |  |  |  |  |
|  | Within prefecture |  | Ref | Ref |  |  |  |
|  | Within province |  |  |  |  |  |  |
|  | Out of province |  | Y(+) | Y(-) |  |  |  |
| Classification | |  |  |  |  |  |  |
|  | PTB smear positive | Y(+) | Y(-) |  | Y(+) |  | Y(+) |
|  | PTB smear negative |  |  |  |  |  |  |
|  | PTB smear unknown |  | Y(+) | Y(+) | Y(+) | Y(+) | Y(+) |
|  | Pleurisy | Ref | Ref | Ref | Ref | Ref | Ref |
|  | EPTB |  |  |  |  |  |  |
| Category | |  |  |  |  |  |  |
|  | New |  |  | Y (-) |  |  |  |
|  | Retreated |  |  | Ref |  |  |  |
| HIV |  |  |  |  |  |  |  |
|  | Positive |  |  |  |  |  |  |
|  | Negative |  | Ref | Ref |  | Ref |  |
|  | Unknown |  | Y(-) | Y(-) |  | Y(-) |  |
| Registered at referral hospital | |  |  |  |  |  |  |
|  | Yes | Ref |  | Ref | Ref |  | Ref |
|  | No | Y(-) |  | Y(-) | Y(-) |  | Y(-) |

*Y – Yes; TB – tuberculosis; PTB – pulmonary tuberculosis; EPTB – exra pulmonary tuberculosis; Ref-reference ; Y(+) positive sign means the factor is an independent predictor for longer delay and Y(-) negative sign means the factor is an independent predictor for shorter delay when compared to reference; *as per web-based TB information management system*
